# The dependence of CO_2_ cerebrovascular reactivity (CVR) on caffeine

**DOI:** 10.1162/IMAG.a.103

**Published:** 2025-08-08

**Authors:** Dinil Sasi Sankaralayam, Cuimei Xu, Zhiyi Hu, Abhay Moghekar, Dengrong Jiang, Chen Hu, Peiying Liu, Hanzhang Lu

**Affiliations:** The Russell H. Morgan Department of Radiology & Radiological Science, Johns Hopkins University School of Medicine, Baltimore, MD, United States; Department of Biomedical Engineering, Johns Hopkins University School of Medicine, Baltimore, MD, United States; Department of Neurology, Johns Hopkins University School of Medicine, Baltimore, MD, United States; Sidney Kimmel Comprehensive Cancer Center, Johns Hopkins University School of Medicine, Baltimore, MD, United States; Department of Diagnostic Radiology & Nuclear Medicine, University of Maryland School of Medicine, Baltimore, MD, United States; F.M. Kirby Center for Functional Brain Imaging, Kennedy Krieger Institute, Baltimore, MD, United States

**Keywords:** cerebrovascular reactivity, caffeine, perfusion, vascular cognitive impairment and dementia, cerebral blood flow

## Abstract

Cerebrovascular reactivity (CVR) represents an important marker of brain vascular health, particularly in the context of small and large vessel diseases. However, an undesired feature of this measure is that there exist large variations in CVR values across individuals, which is mainly attributed to physiological factors. Here, we test the hypothesis that caffeine, a widely consumed neurostimulant, has a significant effect on CVR measured with MRI. Sixteen young healthy participants were enrolled and categorized into caffeine-naive (N = 8) and caffeine-habituated (N = 8) groups based on their caffeine consumption habits. CVR was assessed via CO_2_ inhalation using two different MRI methods, phase-contrast (PC) cerebral blood flow (CBF), and T2*-EPI Blood-Oxygenation-Level-Dependent (BOLD)-MRI. Each participant underwent two MRI sessions, one before and the other after an oral administration of 200 mg of caffeine. Additionally, venous oxygenation (Y_v_) was measured using T_2_-Relaxation-Under-Spin-Tagging (TRUST) MRI. For basal physiological parameters, a significant caffeine-induced CBF decrease was observed in both naive (p = 0.002) and habituated (p < 0.001) groups. The caffeine-naive group exhibited a 31.2 ± 14.1% reduction in basal CBF, whereas the caffeine-habituated group showed a 16.7 ± 5.0% reduction, revealing significant differences between groups (p = 0.04). A similar observation was seen in basal Y_v_, with caffeine-naive participants showing a greater (p = 0.02) reduction (21.5 ± 8.9%) than the habituated participants (7.6 ± 10.1%). CBF-CVR decreased significantly in both groups: from 4.5 ± 0.9 to 3.0 ± 0.9 %CBF/mmHg of CO_2_ (33.3 ± 14.1%, p < 0.001) in the caffeine-naive group, and from 5.1 ± 1.5 to 3.7 ± 1.3 %CBF/mmHg of CO_2_ (27.3 ± 16.0%, p = 0.009) in the caffeine-habituated group. No significant differences were observed between groups in terms of the extent of CVR reduction (p = 0.23). BOLD-CVR showed modest reduction after caffeine administration, from 0.17 ± 0.04 %/mmHg to 0.15 ± 0.05 %/mmHg (14.1 ± 16.8%, p = 0.02). There was no difference between the participant groups in terms of BOLD-CVR reduction following caffeine consumption. This study suggests that investigations using CVR as a disease marker may benefit from accounting for the caffeine consumption and/or its blood concentration in the participants.

## 1 Introduction

Cerebrovascular reactivity (CVR) is a critical measure of the brain’s vascular health, reflecting the capacity of cerebral blood vessels to dilate in response to metabolic demands or vasoactive challenges (Ainslie & Duffin, 2009; Vesely et al., 2001). This dynamic property is essential for maintaining cerebral perfusion and is particularly valuable in the assessment of large vessel diseases, such as arterial stenosis and occlusion, and small vessel diseases, such as vascular cognitive impairment and dementia (VCID) (Glodzik et al., 2013; McKetton et al., 2018; Peng et al., 2018; Sur et al., 2024; Taneja et al., 2020; Thomas et al., 2014). CVR offers a different aspect of insight on vascular health compared with traditional measures of cerebral blood flow (CBF), which provide only a static snapshot of cerebral perfusion.

CVR is typically assessed using MRI through the measurement of hemodynamic responses to a hypercapnia challenge, a state of elevated carbon dioxide (CO_2_) levels that triggers vasodilation (Bhogal et al., 2016; Liu et al., 2019). The resulting CVR index, expressed as the percentage change in MRI signal per mmHg CO_2_, reflects the brain vessel’s responsiveness to changes in CO_2_. Owing to the advances in MRI technologies, CVR imaging has become highly robust in terms of image quality and within-session reproducibility (Liu et al., 2021), suggesting that the thermal noise in the measurement has been effectively reduced. However, there still exist considerable differences in CVR values across individuals and sessions, clouding the quantitative interpretation of CVR and its ability to detect disease-related abnormalities. We hypothesize that these variations are largely attributed to physiological noise or confounding factors, in that different individuals or different sessions may have normal variations in brain physiology, which affect the measured CVR values.

Our previous work has demonstrated that one of such physiological confounding factors is end-tidal CO_2_ (EtCO_2_) of the participant (Hou et al., 2020). Different individuals have slightly different EtCO_2_ levels at rest. It was found that a person who has a higher basal EtCO_2_ tended to have a lower CVR. This information has been used to calibrate CVR in clinical applications to reduce the data variability and improve the sensitivity in disease detection. Nonetheless, even after accounting for EtCO_2_, considerable variations in CVR data remained.

This study aims to investigate the potential role of caffeine as a source of physiological variability in CVR measurements. Caffeine, a widely consumed neurostimulant, induces vasoconstriction by antagonizing adenosine receptors (primarily A_2A_ receptor) located at the vascular smooth muscle cells (vSMCs) (Mishina et al., 2007; O’Regan, 2005; Pelligrino et al., 2010; Svenningsson et al., 1997). Adenosine is a key mediator in hypercapnia-induced vasodilation, where it is produced by endothelial cells and in the extracellular space, binds to A_2A_ receptors on vSMCs to promote relaxation and vasodilation (O’Regan, 2005). This shared molecular pathway between caffeine’s vasoconstrictive effects and hypercapnia-induced vasodilation suggests that caffeine may significantly impact CVR. The literature has extensively documented caffeine’s effect on basal cerebral hemodynamics, including reductions in resting-state CBF (Meno et al., 2005; O’Regan, 2005; Pelligrino et al., 2010; Xu et al., 2015). However, the impact of blood caffeine level on CVR has not been fully elucidated. We hypothesize that caffeine can either decrease CVR, due to reduced availability of adenosine receptors, or increase CVR by providing more room for vasodilation due to a lower basal CBF. This study aims to test this hypothesis in both caffeine-naive and caffeine-habituated healthy individuals by performing CVR MRI measurements before and after the oral administration of a 200 mg caffeine tablet. Two MRI techniques, CBF-MRI using phase-contrast (PC) sequence and Blood-Oxygenation-Level-Dependent (BOLD)-MRI using gradient-echo EPI sequence, were employed as they represent two commonly used hemodynamic parameters for CVR assessment.

## 2 Materials and Methods

### 2.1 Participants

This study included two groups of eight healthy individuals each. The first group consisted of caffeine-naive (Filip et al., 2020; Pickering & Kiely, 2019) individuals who consumed less than 3 cups of coffee per week (equivalent to less than 200 mg of caffeine per week) and comprised 3 males and 5 females (28.6 ± 5.2 years of age). The second group included caffeine-habituated (Filip et al., 2020; Pickering & Kiely, 2019) individuals who consumed at least 4–5 cups of coffee per day (more than 300 mg of caffeine per day) and comprised 5 males and 3 females (29.1 ± 2.7 years of age). Subject recruitment and classification were based on a questionnaire assessing regular caffeine intake (Landrum, 1992). All participants were instructed to refrain from any caffeine consumption after 9 pm the night prior to imaging. The experiments were conducted the next day between 5 pm and 7 pm, ensuring a minimum 20-hour abstinence interval. This study was approved by the institutional review board of the Johns Hopkins University School of Medicine and each subject provided written informed consent before participating in the study.

### 2.2 Study design

All imaging studies were conducted on a Philips Ingenia 3T system (Philips Healthcare, Best, The Netherlands) using a 32 channel receiver head coil.

**Fig. 1.**
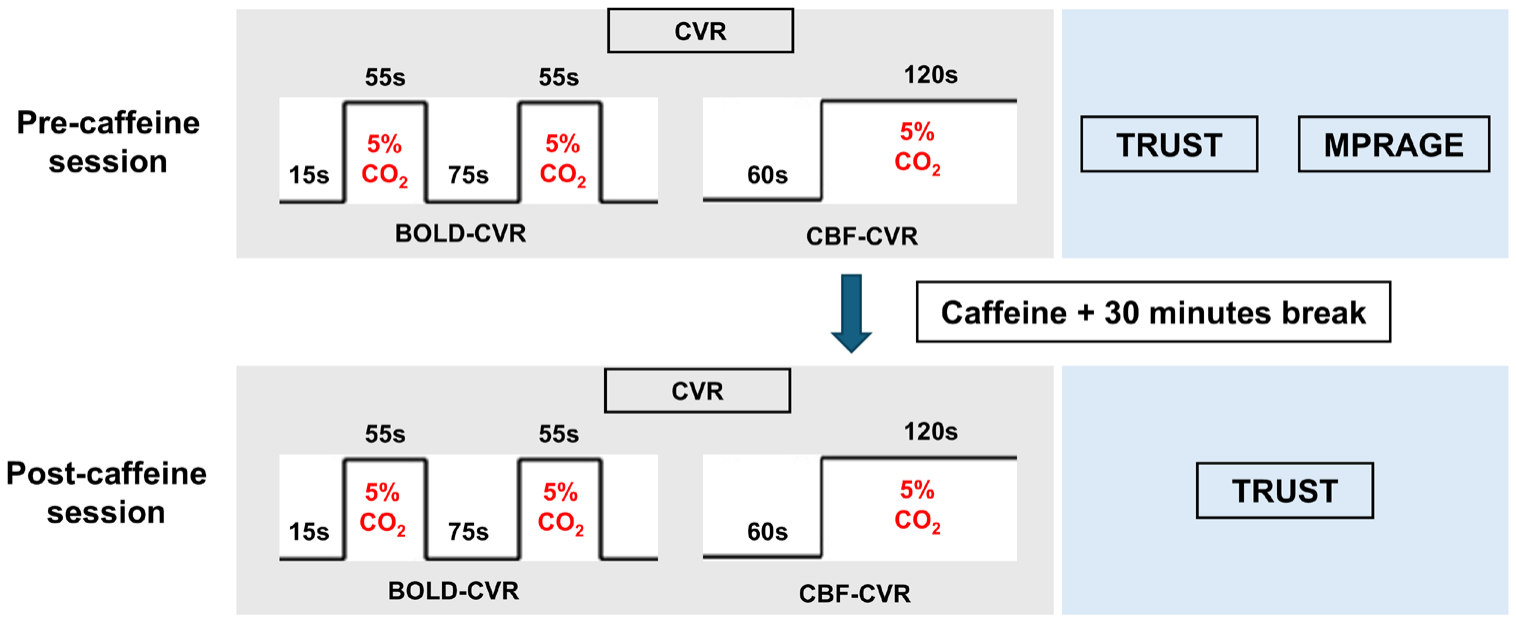
Experimental procedure before and after caffeine administration. The pre-caffeine assessment involved BOLD-MRI and PC-MRI to measure CVR in response to a hypercapnic gas mixture (5% CO_2_, 21% O_2_, 74% N_2_). TRUST MRI was used to evaluate baseline venous oxygenation (Y_v_). MPRAGE was performed to provide a high-resolution T_1_-weighted image to determine whole-brain volume. Participants then ingested a 200 mg caffeine tablet, followed by a 30-minute absorption period. Post-caffeine assessments included repeated measurements of CVR using BOLD-MRI and PC-MRI, as well as reassessment of Y_v_ with TRUST MRI.

The experimental procedure, as illustrated in Figure 1, commenced with a pre-caffeine imaging session. This session included assessments of CVR using both BOLD-MRI and PC-MRI techniques as a readout. PC-MRI provides a quantitative estimation of global changes in blood flow in ml/min, although it does not have spatial information. BOLD-MRI provides a voxel-by-voxel map of CVR, although its signal mechanism is complex and contains several confounding factors. As such, these two CVR techniques are complementary to each other. A T_2_-Relaxation-Under-Spin-Tagging (TRUST) MRI sequence was also performed to evaluate basal venous oxygenation (Y_v_) (without CO_2_ inhalation). Additionally, a high-resolution T_1_-weighted magnetization prepared rapid gradient echo (MPRAGE) structural image was obtained for anatomic reference. Following these baseline measurements, participants were orally administered a 200 mg caffeine tablet, equivalent to approximately 3 cups of coffee. After a 30-minute interval (Z. Lin et al., 2022; Peng et al., 2022; Xu et al., 2015) to allow for caffeine absorption, the CVR and TRUST measurements were repeated to assess the effects of caffeine on these physiological parameters (Fig. 1, bottom panel).

### 2.3 CO_2_-CVR acquisition

The detailed setup for hypercapnia experiment is described elsewhere (Liu et al., 2021). Briefly, each subject was given a mouthpiece for gas inhalation and a nose clip to make sure the subject breathes only through the mouth. A mild hypercapnic air (5% CO_2_, 21% O_2_, and 74% N_2_) stored in a Douglas bag was administered in an interleaved fashion with room air using a valve switch. The BOLD-CVR experiment consisted of an initial room-air phase of 15 s followed by 2 hypercapnic phases of 55 s each, interleaved with room-air phases of 75 s each (Fig. 1). BOLD images and EtCO_2_ were simultaneously measured during the experiment. For CBF-CVR, a single slice PC-MRI was acquired by placing the imaging plane 20 mm above the confluence of sinuses parallel to the AC-PC line. A 1-minute-long PC-MRI was acquired with room-air breathing during the first minute, the valve was then switched to the hypercapnic air, and 2 more PC-MRI were performed. We used first and 3^rd^ PC images for CVR computation, considering that the 2^nd^ PC image was acquired during a transient physiological state. EtCO_2_ measurements during the BOLD-CVR and CBF-CVR scans used a Medtronic capnograph device (Medtronic Capnostream^TM^35 portable respiratory monitor, Oridion Medical 1987 Ltd., Jerusalem, Israel) with a sampling rate of 20 Hz.

The MRI parameters for the BOLD-MRI acquisition were multi-slice 2D EPI, voxel-size = 3.43 × 3.43 × 3.8 mm^3^, flip-angle (FA) = 90^°^, TR/TE = 1500/30 ms, number of dynamics = 200, scan duration = 300 s. The MRI parameters of the PC sequence were voxel-size = 0.4 × 0.4 × 5 mm^3^, TR/TE = 18.7/9.1 ms, number of averages = 4, scan duration = 60 s. We used a Venc = 40 cm/s for room-air acquisition and 60 cm/s for the hypercapnic acquisition, considering the higher blood flow velocity during hypercapnia. To measure the Y_v_, we used a TRUST MRI sequence (Jiang et al., 2018). The imaging parameters for TRUST MRI were single slice voxel size = 3.44 × 3.44 × 5 mm^3^, TR/TE = 3000/3.62 ms, four effective TEs (eTEs) = 0, 40, 80, and 160 ms, and scan duration = 72 s. The parameters for the T_1_-MPRAGE sequence covering the whole brain were voxel-size = 1 × 1 × 1 mm^3^, FA = 12^°^, TR/TE = 8.2/3.78 ms, shot interval = 1934 ms, inversion time = 1100 ms, scan duration = 237 s.

### 2.4 CVR data processing

To compute CBF-CVR, the phase (i.e., velocity) images of the PC scans underwent phase unwrapping if necessary. An ROI was then manually drawn on the complex difference images to delineate the superior sagittal sinus (SSS). This mask was then applied to the velocity maps, and the summation of the voxel-wise values over the ROI yielded the blood flux (BF) in ml/min. Although it does not affect the CVR calculation, for easy comparison with literature values, we further calculated unit-mass CBF (ml/100 g/min) using

$$\text{CBF }=\frac{\text{BF }\left(\frac{\text{ml}}{\text{min}}\right)\times 100}{0.46\;\times \;\text{Bvol }\left(\text{ml}\right)\times 1.06\left(\frac{\text{g}}{\text{ml}}\right)}$$ (1)

where Bvol is the total brain volume (i.e., gray matter + white matter, but not CSF) obtained from T_1_-MPRAGE images using the CAT toolbox (https://neuro-jena.github.io/cat/) (Gaser et al., 2022), 0.46 is the ratio of blood flow in the SSS (De Vis et al., 2018) relative to the total blood flow of the brain, and 1.06 is the mass density of the brain tissue. CBF-CVR was then calculated as Eq. (2).

$$\text{CBF-CVR }=\frac{\frac{{\text{CBF}}_{\text{HC}}-{\text{CBF}}_{\text{RA}}}{{\text{CBF}}_{\text{RA}}}\text{*}100\text{\%}}{\text{EtCO}2_{\text{HC}}-\text{ EtCO}2_{\text{RA}}}$$ (2)

where the subscripts HC and RA represent hypercapnia and room air, respectively, and EtCO_2_ is end-tidal CO_2_ and is in the units of mmHg. CBF-CVR is in units of %/mmHg.

The BOLD-CVR analysis employed a well-established method (Liu et al., 2019) and was performed using a cloud-based online processing tool (https://braingps.mricloud.org/cvr.v4) (Liu et al., 2022). This tool requires BOLD-MRIs and preprocessed EtCO_2_. The detailed pipeline for CVR analysis is described in previously published literature (Liu et al., 2022). Briefly, the BOLD images underwent preprocessing including motion correction, spatial smoothing, and skull stripping using SPM. An automatic envelop detection algorithm identified the EtCO_2_ from the raw CO_2_ recordings, and this curve was time shifted to align with the global BOLD signal. This time-shifted EtCO_2_ curve was then used as a regressor in the general linear model (GLM) with linear trend as a covariate:

$$\text{BOLD }\left(\text{t}\right)=\beta_{0}+\beta_{1}\;\times \;\;\bar{\bar{\text{EtCO}2\left(\text{t},\text{ s}\right)}}\;+\beta_{2}\text{ l},$$ (3)

where BOLD(t) is the BOLD-MRI signal time course, t is time, and β_0_ is the constant, β_1_ is the coefficient associated with the EtCO_2_, β_2_ is the coefficient corresponding to the linear trend, l is the linearly ascending term [- (N - 1)/2, (N + 1)/2,…, (N - 1)/2], where N is the total number of time points, the double bar accent indicates the zero-mean signal, and s is the time shift in EtCO_2_ signal.

BOLD-CVR maps in the units of %/mmHg were then calculated:

$$\begin{array}{l} \text{BOLD}-\text{CVR}\\ \;\;\;\;\;\;\;\;\;=\frac{\beta_{1}}{\beta_{0}\;-\;\beta_{1}\times \left(\text{mean}\left(\text{EtCO}2\right)-\text{baseline }\left(\text{EtCO}2\right)\right)}\text{*}100, \end{array}$$ (4)

where mean (EtCO_2_) and baseline (EtCO_2_) are the mean and bottom 25% of the EtCO_2_ values. With the calculation in Eq. (4), the BOLD-CVR index indicates the change of BOLD signal from the room-air state, rather than from a virtual state of EtCO_2_ = 0 mmHg, as detailed previously (Liu et al., 2022). The CVR map was then co-registered to the MPRAGE image and normalized to the MNI space. Regional masks corresponding to different brain areas were generated using a cloud-based online processing tool (https://braingps.mricloud.org/t1prep) (Mori et al., 2016) and applied to the BOLD-CVR maps to compute ROI values including the whole brain, whole-brain gray matter, whole-brain white matter, frontal lobe gray matter, temporal lobe gray matter, parietal lobe gray matter, occipital lobe gray matter, limbic lobe, insula, thalamus, and basal ganglia.

### 2.5 TRUST MRI processing

TRUST MRI processing was performed using the previously described procedure (Jiang et al., 2018; Lu et al., 2012). Briefly, a pair-wise subtraction of label and control images was performed after motion correction to yield signals from pure venous blood at the SSS. An ROI surrounding the SSS was manually drawn, and the algorithm automatically detects the top 4 highest intensity voxels from the ROI. These values at each eTE were spatially averaged and fitted using a mono-exponential model as a function of eTE. The estimated blood T_2_ was then converted to Y_v_ using a previously established calibration model (Lu et al., 2012) to derive subject-specific Y_v_ values using literature-reported hematocrit (Hct) values (Hct = 0.4 for female, Hct = 0.42 for male).

### 2.6 Statistical analysis

All statistical analyses were performed using MATLAB (version R2022a, The MathWorks Inc., Natick, MA, USA) and the Statistics and Machine Learning Toolbox.

#### 2.6.1 Between-group comparison of baseline physiology

First, independent t-tests were conducted to evaluate the pre-caffeine physiological measures (basal CBF, basal Y_v_, CBF-CVR, and BOLD-CVR) between the caffeine-naive and caffeine-habituated groups, prior to caffeine administration. The goal was to assess whether any significant group-level differences existed at baseline.

#### 2.6.2 Within-subject comparison of pre- and post-caffeine basal measures

Paired t-tests were then performed to assess the within-subject changes in basal CBF, basal Y_v_, CBF-CVR, and BOLD-CVR before and after caffeine administration. This analysis aimed to determine the significance of caffeine’s effect on each physiological measure. The assumption of normality for the differences in pre- and post-caffeine values was evaluated using the Shapiro–Wilk test.

#### 2.6.3 Linear mixed-effects model analysis

Lastly, a linear mixed-effects model was employed to further assess the effect of caffeine on the outcome measures (basal CBF, basal Y_v_, CBF-CVR, and BOLD-CVR), while controlling for additional covariates. The model was specified as follows:

$$\begin{array}{l} \text{Outcome}\;\sim \;\text{Caffeine Status * Group}\;+\;\left(1\;\text{|}\;\text{Subject ID}\right)\\ +\;\text{Covariates}+\epsilon , \end{array}$$

where the outcome is the dependent variable (basal CBF, basal Y_v_, CBF-CVR, or BOLD-CVR), caffeine status is a binary fixed effect indicating pre- (0) or post- (1) caffeine administration, group is a binary fixed effect (caffeine-naive or caffeine-habituated), sex and age are included as covariates, caffeine status × group represents the interaction term, (1|Subject ID) accounts for subject-specific variability through random intercepts, ϵ represents the residual error term.

## 3 Results

### 3.1 Representative MR images and EtCO_2_ recordings

**Fig. 2.**
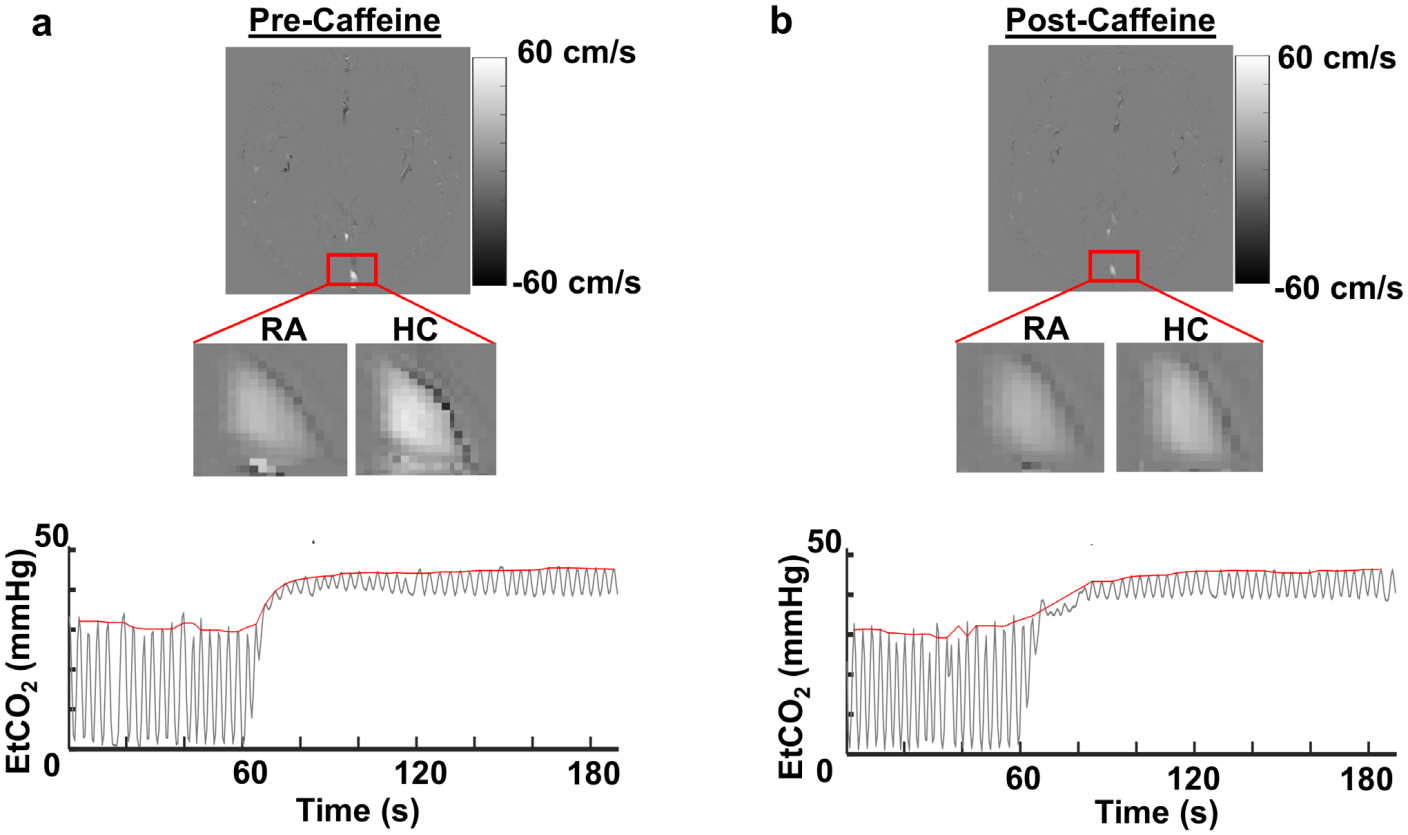
Phase-contrast flow velocity images under room-air (RA) and hypercapnia conditions (HC), along with end-tidal CO_2_ (EtCO_2_) recordings. (a) Before caffeine administration. (b) After caffeine administration. EtCO_2_ curves illustrate the CO_2_ levels during pre-caffeine and post-caffeine conditions. Note that the velocity images became brighter during HC in comparison with RA, suggesting an increased blood flow.

Figure 2 presents phase-contrast velocity maps and corresponding EtCO_2_ recordings from a representative caffeine-naive participant, both before (Fig. 2a) and after (Fig. 2b) caffeine administration. For each condition, zoomed-in views of phase images during room air (RA) and hypercapnia (HC) are also displayed. The EtCO_2_ recordings in the figure demonstrate an increase due to CO_2_ inhalation, which corresponds to changes in blood flow depicted in the respective phase images. EtCO_2_ values obtained from both groups are given as Supplementary Table S1.

### 3.2 Effect of caffeine on basal CBF and Y_v_

**Fig. 3.**
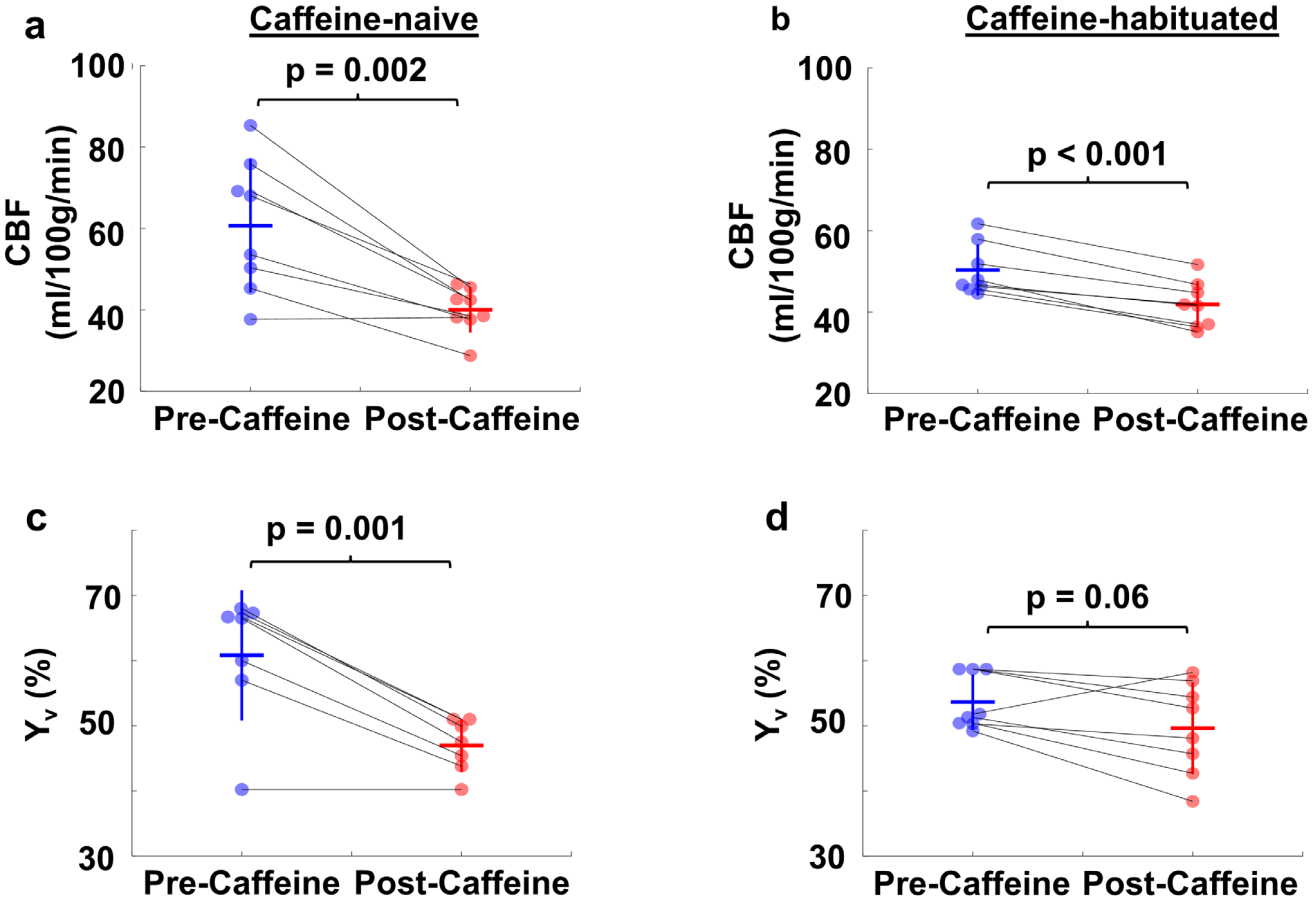
Impact of caffeine administration on basal cerebral blood flow (CBF) and venous oxygenation (Y_v_) in both caffeine-naive and caffeine-habituated participants. (a, b) Basal CBF values pre- and post-caffeine administration. (c, d) Basal Y_v_ pre- and post-caffeine administration. Purple dots represent pre-caffeine status and red dots represent post-caffeine status.

Basal CBF was not different between caffeine-naive and caffeine-habituated participants (p = 0.12). Caffeine administration resulted in a significant reduction in basal CBF in both the caffeine-naive (p = 0.002) and caffeine-habituated groups (p < 0.001), as depicted in Figure 3a and b. In the caffeine-naive group, caffeine administration resulted in a 31.2 ± 14.1% (mean ± standard deviation) reduction in basal CBF, while the caffeine-habituated group showed a 16.7 ± 5% reduction, exhibiting a significant difference between groups (p = 0.04). A mixed-effect model analysis with age, sex, group, and caffeine state as fixed effects and subject ID as random effect revealed a significant sex effect (β = -9.5 ml/100 g/min, 95% CI -16.4 to -2.73, p = 0.008), caffeine effect (β = -20.7 ml/100 g/min, 95% CI -26.9 to -14.39, p < 0.0001), and interaction effect between caffeine state and group (β = 12.2 ml/100 g/min, 95% CI 3.4 to 21.1, p = 0.009). Specifically, the effect of caffeine on basal CBF and change in CBF due to caffeine were more pronounced in the caffeine-naive group than in the caffeine-habituated group.

Similar to basal CBF, basal Y_v_ did not exhibit a group effect (p = 0.08). In the caffeine-naive group, Y_v_ values decreased from 60.8 ± 9.2% to 46.9 ± 3.7% (p = 0.001), a reduction by 21.5 ± 8.9% (Fig. 3c). In contrast, the Y_v_ change in the caffeine-habituated group was not statistically significant (p = 0.06), with values decreasing from 53.6 ± 3.9% to 49.6 ± 6.6%, representing a reduction of 7.6 ± 10.1% (Fig. 3d). A linear regression of ΔY_v_ on age, sex, and group revealed a significant group effect (p = 0.02), confirming that naive participant’s Y_v_ reduction exceeded habituated participants. In the mixed-effects model, baseline Y_v_ was associated with sex (β = -5.9%, 95% CI -11.7 to -0.2, p = 0.045). Caffeine intake (post vs. pre) reduced Y_v_ by 13.7% on average (β = -13.7%, 95% CI -17.9 to -9.5, p < 0.001). Moreover, the caffeine status × group interaction was significant (β = 9.7%, 95% CI 4.0 to 15.4, p = 0.0019), indicating that habituated subjects’ Y_v_ drop was markedly smaller than naive subjects’ drop. That is, consistent with the CBF findings, the effect of caffeine on basal venous oxygenation was more pronounced in the caffeine-naive group than in the caffeine-habituated group.

### 3.3 Effect of caffeine on CBF-CVR

**Fig. 4.**
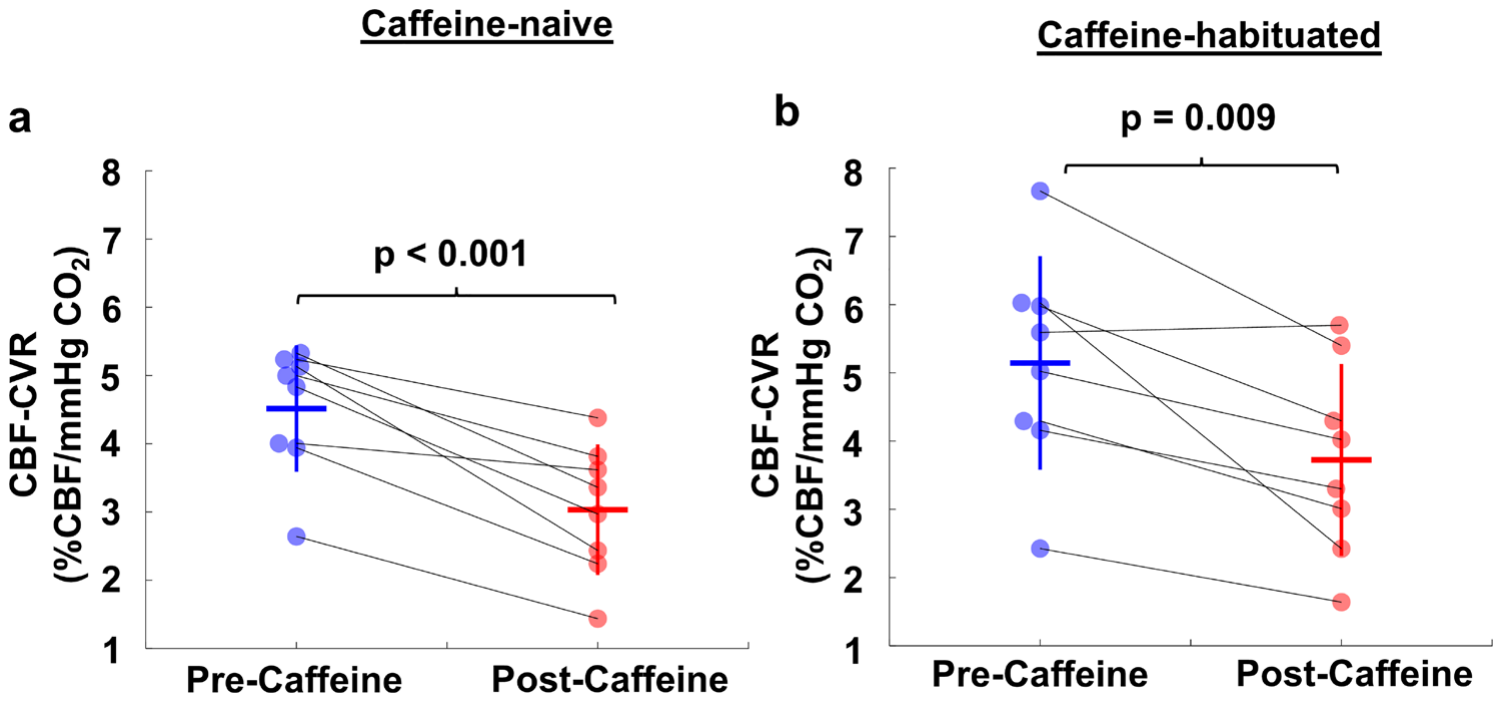
CBF-CVR values before and after caffeine ingestion. (a) Caffeine-naive participants. (b) Caffeine-habituated participants.

Figure 4 illustrates CBF-CVR results in both caffeine-naive (Fig. 4a) and caffeine-habituated groups (Fig. 4b). We did not observe a significant difference in pre-caffeine CVR with age, sex, or group. Both groups exhibited a significant reduction in CBF-CVR following caffeine administration. In the caffeine-naive group, CBF-CVR decreased from 4.5 ± 0.9 to 3.0 ± 0.9 %/mmHg of CO_2_, corresponding to a reduction of 33.3 ± 14.1% (p < 0.001). The caffeine-habituated group showed a reduction in CBF-CVR from 5.1 ± 1.5 to 3.7 ± 1.3 %/mmHg of CO_2_, corresponding to a decrease by 27.3 ± 16.0% (p = 0.009). However, this difference was not significantly different between groups. The linear mixed-effect model revealed that there is a significant caffeine effect (β = -1.5 %CBF/mmHg CO_2_, 95% CI -2.1 to -0.8, p < 0.001), however, there was not a caffeine state by group interaction effect (p = 0.89), suggesting that the effect of caffeine ingestion on CVR was similar between the caffeine-naive and caffeine-habituated individuals.

### 3.4 Effect of caffeine on BOLD-CVR

**Fig. 5.**
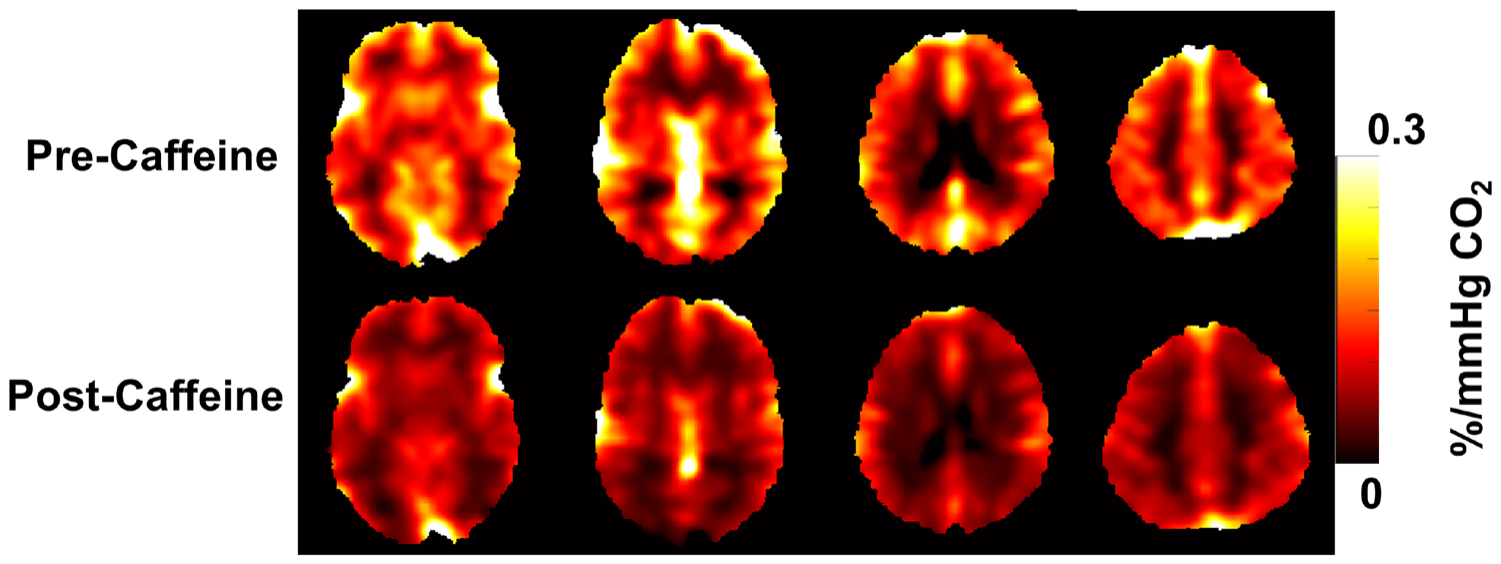
Representative BOLD-CVR maps from a caffeine-naive subject before and after caffeine administration.

**Fig. 6.**
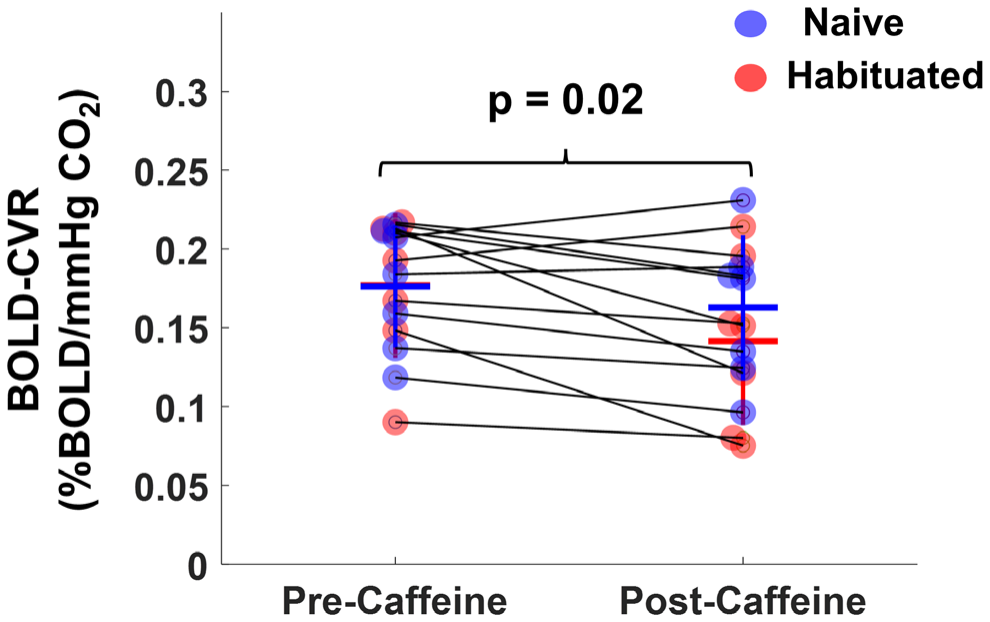
Whole-brain BOLD-CVR values before and after caffeine ingestion combining both naive and habituated participants.

Before conducting the statistical analysis, BOLD-CVR values were adjusted for baseline end-tidal CO_2_ (bEtCO_2_) and the change in end-tidal CO_2_ (ΔEtCO_2_) in accordance with a previous study (Hou et al., 2020; Sur et al., 2024). Figure 5 shows representative BOLD-CVR maps from a caffeine-naive subject before and after caffeine administration. There was not a significant difference in baseline BOLD-CVR between the caffeine-naive and caffeine-habituated groups. Additionally, caffeine administration did not result in a significant change in whole-brain BOLD-CVR within either group. However, when combining data from both groups, post-caffeine whole-brain BOLD-CVR values showed a modest reduction of 14.1 ± 16.8%, decreasing from 0.17 ± 0.04 to 0.15 ± 0.05 %/mmHg (p = 0.02) as shown in Figure 6. Regional analysis indicated that BOLD-CVR significantly decreased in the parietal lobe (p = 0.04), occipital lobe (p = 0.01), and basal ganglia (p = 0.02) following caffeine administration (Supplementary Table S2).

## 4 Discussion

This study investigated an important source of variations related to caffeine in CVR MRI data. We found that CVR was diminished after caffeine intake. This reduction was observed when using both CBF-based CVR technique and BOLD-based CVR technique, although the percentage of reduction was greater for CBF-CVR (~30%) than for BOLD-CVR (~15%). Importantly, these effects were consistent in both caffeine-naive and caffeine-habituated healthy participants. Additionally, analyses of basal physiological parameters (under room-air conditions without CO_2_ inhalation) demonstrated that caffeine reduced both basal CBF and Y_v_, with more pronounced effects in the caffeine-naive group than in the caffeine-habituated group.

### 4.1 CVR as a potential diagnostic marker: The significance of understanding the sources of its physiological variations

CVR is increasingly recognized as a valuable biomarker in brain diseases including cerebrovascular diseases but also diseases of non-vascular origin (van der Horn et al., 2024; Wang et al., 2024). For example, recent research has highlighted the relationship between CVR measures and various aspects of cognitive and physical function, independent of Alzheimer’s disease pathologies such as tau, phosphorylated tau, and amyloid-beta (Aβ) (Sur et al., 2020, 2024; Taneja et al., 2020). However, like other physiological measures such as CBF and cerebral blood volume, one lingering concern that plaques CVR as a disease biomarker is its relatively large variations across individuals and sessions. It should be emphasized that these variations are not due to intrinsic signal-to-noise (SNR) limitations of MRI. In fact, CO_2_-induced BOLD-MRI signal change is greater than the strongest functional stimulus (e.g., flashing checkerboard in the visual cortex) in fMRI studies; thus thermal noise is no longer a major issue in CVR MRI. Instead, the variations observed in CVR data are primarily attributed to factors we generally term as “physiological noise” (Golestani & Chen, 2020). Physiological noise can have many sources, ranging from respiratory and cardiac to dietary factors. Once we can understand and correct the impact of these confounding factors, normal variations in CVR will be substantially reduced and CVR has the potential to become a highly sensitive and personalized disease biomarker.

Different individuals (even healthy ones) have slightly different EtCO_2_ levels at rest. From a group of 253 healthy subjects we studied, we found a mean EtCO_2_ of 38.1 mmHg and a standard deviation of 4.3 mmHg (Hou et al., 2020). We showed that the basal EtCO_2_ represents a significant source contributing to normal variations in BOLD-CVR. For every unit of differences in EtCO_2_, CVR varies by approximately 2%. A similar effect was observed for the change in EtCO_2_ due to CO_2_ inhalation (ΔEtCO_2_). For every unit of difference in ΔEtCO_2_, CVR varies by approximately 4%. Thus, much of the CVR variations can be explained by EtCO_2_ differences. However, there remains a significant amount of variations in CVR after accounting for EtCO_2_. Thus, other physiological sources need to be identified.

### 4.2 Effect of caffeine on CVR

Caffeine, a widely consumed psychoactive substance, exerts intricate effects on cerebral vessels in addition to its influence on alertness and neuronal activity. Its primary mechanism of action on vasculature involves antagonism of adenosine receptors, particularly the A_2A_ subtype, which plays a crucial role in regulating cerebral blood flow (Evatt & Griffiths, 2013; Pelligrino et al., 2010). Adenosine is a potent vasodilator and promotes increased vascular tone by acting on vSMCs. By blocking adenosine receptors, caffeine can reduce vascular tone, leading to a reduction in CBF (Koppelstaetter et al., 2010; Pelligrino et al., 2010). A_2A_ receptors are predominantly found in vSMCs and are also involved in the vasodilatory effects during hypercapnia. Therefore, the pathways of caffeine and hypercapnia actions intersect.

One may hypothesize two possible outcomes in terms of the effect of caffeine on CVR. On the one hand, caffeine’s reduction of basal vascular tone and blood flow could theoretically allow greater capacity for blood vessels to dilate in response to hypercapnia, potentially increasing CVR. On the other hand, caffeine’s antagonism of adenosine receptors might restrict vasodilation during hypercapnia, leading to a decrease in CVR. The results of our study demonstrated a significant reduction in CVR following caffeine administration, indicating a diminished vascular response to hypercapnic stimuli and supporting the latter hypothesis. This suggests that the availability of adenosine receptors, rather than the presence of adenosine itself, is the rate-limiting factor in hypercapnia-induced vasodilation.

This study also reproduced the effects of caffeine on basal physiological parameters that have been reported previously. For example, we found that caffeine ingestion significantly reduced basal CBF (Gaspar et al., 2024; Z. Lin et al., 2022; Xu et al., 2015). Similarly, Y_v_ was also decreased by caffeine. These effects are due to caffeine’s potent vasoconstrictive properties through adenosine receptor blockade.

Three previous studies have investigated the impact of acute caffeine ingestion on vascular responses to CO_2_. Peng et al. (2022) administered 200 mg of caffeine to habitual and non-habitual volunteers and found significant reductions in basal CBF. While the investigation did not measure end-tidal CO_2_, they used a breath-hold challenge to measure BOLD signal changes before and after caffeine administration. It was found that BOLD response was diminished in the naive group but not in habitual individuals (Peng et al., 2022). Chen and Parrish (2009) applied a calibrated‐BOLD approach-combining 5% CO_2_ inhalation with simultaneous CBF and BOLD monitoring during motor and visual tasks—to estimate the CBF:CMRO_2_ coupling ratio (n). With a 2.5 mg/kg IV caffeine dose, they saw a significant decrease in n from 2.58 to 2.33 in motor cortex and from 2.45 to 2.23 in visual cortex, yet CO_2_‐driven BOLD‐CVR was unaffected. Blaha et al. (2007) recorded middle cerebral artery velocities via transcranial Doppler before and 30 minutes after a 300 mg caffeine dose, observing significant decreases in resting and hypercapnic velocities but no change in CO_2_ reactivity. The present study measured end-tidal CO_2_ and obtained quantitative CVR, using both CBF and BOLD sequences as readouts. Our findings are similar to the BOLD results of Peng et al. (2022), in that caffeine reduces CVR. However, we did not observe a group difference between naive and habituated participants.

### 4.3 CBF-CVR versus BOLD-CVR

Several MRI techniques can be employed for CVR measurement, each with its advantages and limitations. BOLD-MRI is the most widely used acquisition sequence. However, its physiological underpinning is complex, with its signal related to multiple physiological parameters rather than a single one (Catchlove et al., 2018; Liu et al., 2013, 2019, 2021). CBF MRI sequences provide a measure that is directly related to cerebral blood flow, thus is easy to interpret, although these sequences either have low signal-to-noise ratio (SNR) or have limited spatial resolution (Sur et al., 2024; Taneja et al., 2020). In this study, we acquired CVR using two different techniques, a global CBF-CVR using a phase-contrast quantitative flow sequence and a voxel-by-voxel BOLD-CVR using a BOLD sequence.

We found that the effect of caffeine on CVR was more pronounced when calculated based on CBF, which showed a reduction by approximately 30%. In contrast, caffeine-induced diminishment on BOLD-CVR was only about 15%. This difference can be explained by the BOLD signal mechanism. Specifically, the BOLD signal change during hypercapnic gas inhalation can be written as (Bulte et al., 2012; Gauthier & Fan, 2019) $\frac{\Delta {\text{BOLD}}_{\text{HC}}}{{\text{BOLD}}_{0}}\alpha {\text{ CBV}}_{\text{0}}{\left[\text{dHb}\right]}_{0}^{\beta }(1-{\left(\frac{{\text{CBF}}_{\text{HC}}}{{\text{CBF}}_{0}}\right)}^{\propto -\beta }$ , where CBF_HC_ and CBF_0_ represent CBF values during hypercapnia and room air, respectively, CBV_0_ is the baseline venous cerebral blood volume, [dHb]_0_ is the baseline deoxyhemoglobin concentration, and α is the power-law co-efficient and β accounts for the effect of voxel size. By analyzing the directionality of the terms in the above equation, it can be seen that the expected reduction in BOLD-CVR due to diminished CBF response is counteracted by an increase in [dHb]_0_, which is related to 1-Y_v,0_. Thus the changes in basal venous oxygenation partly offset the CVR reduction when using the BOLD technique.

Moreover, our regional analyses revealed that parietal lobe, occipital lobe, basal ganglia, and putamen showed the most pronounced caffeine‐induced BOLD‐CVR reduction. This pattern likely reflects two converging factors. First, subcortical nuclei such as the basal ganglia and putamen are known to express especially high levels of adenosine A_2A_ receptors (Schiffmann et al., 2007), thus caffeine’s antagonism produces strong vasoconstriction there. Second, parietal and occipital cortices are known to have a lower basal venous oxygenation (Krishnamurthy et al., 2014) (i.e., higher deoxyhemoglobin). Thus, these regions tend to have more robust BOLD signal, affording a higher sensitivity in detecting a caffeine effect on BOLD-CVR.

### 4.4 Caffeine-naive versus caffeine-habituated participants

It is reasonable to expect that daily caffeine consumption varies considerably among research participants. We, therefore, specifically enrolled two groups of participants in this study, a caffeine-naive group consisting of individuals who consumed less than 3 cups of coffee per week and the other group of caffeine-habituated individuals who consumed at least 4–5 cups of coffee per day. We found that CBF-CVR was reduced after caffeine ingestion in both participant groups and the extent of decrease was similar (~30%), suggesting that the effect of caffeine on CVR was relatively independent of daily caffeine consumption.

In contrast, when examining the impact of caffeine ingestion on basal physiological parameters, we found a more pronounced effect in caffeine-naive individuals than in caffeine-habituated individuals. For example, caffeine ingestion reduces basal CBF (without CO_2_ inhalation) by 31.2% in caffeine-naive participants while this value was only 16.7% in caffeine-habituated participants. A similar pattern was observed in Y_v_, exhibiting a great effect in caffeine-naive subjects. These findings suggest that caffeine-habituated individuals may have a reduced density of adenosine receptors, or their receptors have a lower affinity of binding to caffeine molecules, rendering them less susceptible to caffeine’s antagonistic effects on adenosine‐mediated vasodilation. Furthermore, since our caffeine-naive and habituated groups are slightly unbalanced in terms of their male and female distributions, we also repeated the statistical analyses separately for each of the sex sub-groups. The findings from the sub-group analyses were similar to that using both sexes.

It was also noted that the variation in basal CBF among the caffeine-naive participants (15.4 ml/100 g/min) was greater than that of the habituated group (5.9 ml/100 g/min). This could be because the caffeine-naive participants had a larger variation in their end-tidal CO_2_ (4.5 mmHg) than the habituated group (3.2 mmHg).

### 4.5 Clinical implications

The observed effects of caffeine on CVR measurements underscore the importance of accounting for caffeine influence in research and clinical CVR studies. Due to heterogeneities in daily caffeine consumption across individuals, normal variations in CVR may be amplified. By understanding the influence of caffeine on CVR and potentially correcting it (e.g., via the measurement of plasma concentration of caffeine), one may be able to reduce its contribution to physiological noise in CVR data, thereby enhancing the detection of disease state. Similarly, when applying CVR to clinical trials as a marker to index the drug effect in terms of improving microvascular function, controlling caffeine effects may reduce the necessary trial sample size.

### 4.6 Limitations and future directions

Our study has several limitations. The sample size of this study is relatively small. A larger sample size may allow the observation of a significant effect on BOLD-CVR in each of the caffeine-naive and habituated groups. We also note that the main goal of the present study is to characterize the effect of caffeine on CVR. Our study is not sufficiently powered to definitely determine the impact of daily caffeine consumption on the caffeine–CVR relationship. Furthermore, study timing has to be considered more carefully if one aims to fully elucidate how habituated participants respond to acute caffeine intake. It is anticipated that the change in CBF (and related physiological parameters) after the cessation of daily caffeine intake follows a complex, non-monotonic time course (Field et al., 2003; Y.-S. Lin et al., 2022). In the first few hours after stopping consuming caffeine, there will be residual effects from caffeine and its primary metabolite paraxanthine, resulting in a CBF reduction (Y.-S. Lin et al., 2022; Lin et al., 2021). After the residual effects are dissipated, there are possible withdrawal effects. Some studies have shown an elevated CBF after withdrawal from daily caffeine intake (Field et al., 2003; Jones et al., 2000; Lin et al., 2021). This effect has been reported to last until 36 hours after the cessation of caffeine consumption. The results from the present study were only measured at a single time point (approximately 20 hours after cessation), thus should only be viewed as a snapshot of a more complex physiological response. A future multi-time-point study is needed to fully characterize this response.

The current study focused solely on within-subject effects of caffeine. A further investigation is needed to elucidate how inter-subject variability in CVR can be explained by caffeine blood concentration. Additionally, future research should examine whether the observed effect of caffeine varies between healthy individuals and patients with cerebrovascular diseases. Finally, we note that we did not collect participants’ body weight data, and, therefore, could not normalize the 200 mg caffeine dose by body mass. Since lighter or heavier individuals may achieve different plasma caffeine concentrations for a fixed oral dose, the absence of weight‐normalized dosing represents a limitation. Future studies should record body weight (and ideally lean body mass) so that caffeine’s effects on both baseline hemodynamics and CVR can be examined on a mg/kg basis, minimizing potential confounding by inter‐individual differences in caffeine exposure.

## 5 Conclusion

This study demonstrates that caffeine has a significant effect on CVR as measured with MRI and CO_2_ inhalation. This effect was present in both caffeine-naive and habituated individuals. The effect size appears to be dependent on the MRI acquisition approach used, with CBF-CVR revealing a greater extent of reduction than BOLD-CVR. This study also observed that caffeine reduced basal CBF and Y_v_, consistent with its vasoconstrictive effects. Future studies using CVR as a disease marker may benefit from accounting for the caffeine consumption and/or its blood concentration in the participants.

## Supplementary Material

Supplementary Material

## Data Availability

Data and code are available upon request.
