# Supplementary material for: The dependence of CO2 cerebrovascular reactivity (CVR) on caffeine
Source: Imaging Neurosci (Camb). 2025 Aug 8;3:IMAG.a.103. doi: 10.1162/IMAG.a.103 (PMC12336059; doi:10.1162/IMAG.a.103)
Supplement: Supplementary Material [file IMAG.a.103_supp.pdf]

Supplementary Tables

			Naïve	Habituated
**$EtCO_2$ (CBF-CVR) (mmHg CO_2)**	**Pre-Caffeine**	**$EtCO_2$ (RA)**	36.7±3.2	37.3±4.5
		**$EtCO_2$ (HC)**	47.4±2.0	47.8±4.5
		**$\Delta EtCO_2$**	10.7±2.5	10.4±2.0
	**Post-Caffeine**	**$EtCO_2$ (RA)**	34.9±2.4	35.6±3.6
		**$EtCO_2$ (HC)**	45.7±1.6	46.0±4.2
		**$\Delta EtCO_2$**	10.9±2.0	10.4±2.1
**$EtCO_2$ (BOLD-CVR) (mmHg CO_2)**	**Pre-Caffeine**	**$EtCO_2$ (RA)**	35.7±2.9	37.3±5.1
		**$EtCO_2$ (HC)**	45.6±2.6	46.5±5.0
		**$\Delta EtCO_2$**	9.9±2.9	9.2±2.8
	**Post-Caffeine**	**$EtCO_2$ (RA)**	34.5±2.1	34.9±4.0
		**$EtCO_2$ (HC)**	45.1±2.2	45.5±5.1
		**$\Delta EtCO_2$**	10.6±2.3	10.5±2.8

Supplementary Table 1: End-tidal CO_2 ($EtCO_2$) measurements during room-air (RA) and hypercapnia (HC) phases for both CBF-CVR and BOLD-CVR scans in caffeine-naïve and caffeine-habituated participants, before and after caffeine. Values are mean ± SD in mmHg CO_2. $\Delta EtCO_2$ denotes the change in $EtCO_2$ between HC and RA periods (HC – RA).

	Pre-Caffeine	Post-Caffeine	p-value
**Whole Brain**	0.17±0.04	0.15±0.05	**0.02**
**Gray Matter**	0.18±0.07	0.16±0.06	0.07
**White Matter**	0.12±0.05	0.12±0.04	0.28
**Frontal**	0.18±0.07	0.16±0.06	0.13
**Temporal**	0.20±0.07	0.18±0.07	0.18
**Parietal**	0.18±0.07	0.15±0.06	**0.04**
**Occipital**	0.19±0.06	0.16±0.06	**0.01**
**Limbic**	0.17±0.07	0.15±0.06	0.51
**Insula**	0.15±0.07	0.13±0.05	0.32
**Thalamus**	0.19±0.07	0.18±0.07	0.23
**Basal Ganglia**	0.14±0.05	0.11±0.04	**0.02**

Supplementary Table 2: Regional BOLD-CVR values (mean ± SD; % BOLD/mmHg CO_2) in the whole brain and major anatomical regions, measured before after caffeine. p values are from paired t-tests assessing within-subject pre- vs. post-caffeine differences; values in bold indicate $p<0.05$.
